# Supplementary material for: Analysis of PPARγ Signaling Activity in Psoriasis
Source: Int J Mol Sci. 2021 Aug 10;22(16):8603. doi: 10.3390/ijms22168603 (PMC8395241; doi:10.3390/ijms22168603)
Supplement: Supplementary file 1 [file ijms-22-08603-s001.zip › Supplemental materials_Analysis of PPARg signaling activity in psoriasis/Pathway models/Models images and html files/PPARG inhibits phenotypes assotiated with psoriasis/122738.html]

kidney disease


# Disease kidney disease

|  |  |
| --- | --- |
| URN | urn:agi-meshdis:Kidney%20Diseases |
| Total Entities | 59 |
| Connectivity | 6242 |
| Name | kidney disease |

---

|  |  |
| --- | --- |
| ChildConcepts | renal neoplasm |
|  | glomerular necrosis |
|  | renal failure |
|  | nephritis |
|  | renal colic |
|  | analgesic nephropathy |
|  | renal abnormality |
|  | renal dysfunction |
|  | glomerulopathy |
|  | distal arthrogryposis type 3 |
|  | renal infection |
|  | renal allograft dysfunction |
|  | renal amyloidosis |
|  | renovascular disease |
|  | renal cyst |
|  | renal hemorrhage |
|  | renal injury |
|  | renal necrosis |
|  | renal pain |
|  | renal rupture |
|  | renal scarring |
|  | renal tubule damage |
|  | perinephric abscess |
|  | tubulopathy |
|  | pyelectasis |
|  | reflux nephropathy |
|  | silent kidney |
|  | uric acid nephropathy |
|  | renal hemangiomyolipoma |
|  | diabetic nephropathy |
|  | nephrosis |
|  | rare renal disease |
|  | podocyte dysfunction |
|  | polyomavirus-associated nephropathy |
|  | cystic kidney disease |
|  | interstitial fibrosis |
|  | tubular degeneration |
|  | hepatorenal syndrome |
|  | renal papillary necrosis |
|  | perinephritis |
|  | renal artery obstruction |
|  | HIV-associated nephropathy |
|  | glomerular infiltration |
|  | nephroptosis |
|  | Lachiewicz-Sibley syndrome |
|  | urine concentrating defect |
|  | renal atrophy |
|  | nephrocalcinosis |
|  | nephrolithiasis |
|  | renal hyperfiltration |
|  | renal infiltration |
|  | renal ischemia |
|  | nephrotoxicity |
|  | glomerular deposition |
|  | glomerular hyperfiltration |
|  | glomerular hypercellularity |
|  | renal infarction |
|  | nephroangiosclerosis |
|  | renal complication |

---

|  |  |
| --- | --- |
| Pathway | PPARG inhibits phenotypes assotiated with psoriasis |
|  | Neighbors of kidney disease |

---

|  |  |
| --- | --- |
| MedScan ID | 9001703 |

---

|  |  |
| --- | --- |
| Alias | radiocontrast nephropathies |
|  | Kidney Diseases |
|  | kidney symptoms |
|  | Nephropathies |
|  | kidney syndromes |
|  | glomerulus disorders |
|  | Disorder of Kidney |
|  | obstructive nephropathy |
|  | renal pathologies |
|  | Disorder Renal |
|  | pathology in the kidney |
|  | Diseases, Kidney |
|  | impairments in renal function |
|  | kidney deterioration |
|  | Kidney Disorders |
|  | Nephropathy |
|  | renal symptoms |
|  | proximal nephropathy |
|  | kidney pathology |
|  | Kidney Disease |
|  | renal deteriorations |
|  | Nephrologic Disease |
|  | unilateral kidney diseases |
|  | pathogenesis of kidney |
|  | renal pathogeneses |
|  | kidney syndrome |
|  | impairment of renal function |
|  | disorders of the kidney |
|  | glomerulus disease |
|  | renal function impairment |
|  | Renal Disorder |
|  | renal function impairments |
|  | renal pathogenesis |
|  | Renal Disease |
|  | Renal Disorders |
|  | obstructive nephropathies |
|  | kidney pathogenesis |
|  | kidney pathologies |
|  | radiocontrast nephropathy |
|  | glomerulus pathogenesis |
|  | renal problems |
|  | deterioration of kidney |
|  | Renal Diseases |
|  | renal deterioration |
|  | glomerulus diseases |
|  | renal event |
|  | unilateral kidney disease |
|  | glomerulus pathology |
|  | Kidney Disorder |
|  | renal impairment |
|  | Disease, Kidney |
|  | pathologies in the kidney |
|  | Nephrologic Diseases |
|  | renal pathology |
|  | renal impairments |
|  | renal events |
|  | proximal nephropathies |

---

|  |  |
| --- | --- |
| MeSH ID | D007674 |
|  | M0012014 |

---

|  |  |
| --- | --- |
| MeSH Heading | Kidney Diseases |

---
